# The comparative analysis of SARIMA, Facebook Prophet, and LSTM for road traffic injury prediction in Northeast China

**DOI:** 10.3389/fpubh.2022.946563

**Published:** 2022-07-22

**Authors:** Tianyu Feng, Zhou Zheng, Jiaying Xu, Minghui Liu, Ming Li, Huanhuan Jia, Xihe Yu

**Affiliations:** School of Public Health, Jilin University, Changchun, China

**Keywords:** road traffic injuries, time series analysis, machine learning, predictive models, comparative study

## Abstract

**Objective:**

This cross-sectional research aims to develop reliable predictive short-term prediction models to predict the number of RTIs in Northeast China through comparative studies.

**Methodology:**

Seasonal auto-regressive integrated moving average (SARIMA), Long Short-Term Memory (LSTM), and Facebook Prophet (Prophet) models were used for time series prediction of the number of RTIs inpatients. The three models were trained using data from 2015 to 2019, and their prediction accuracy was compared using data from 2020 as a test set. The parameters of the SARIMA model were determined using the autocorrelation function (ACF) and the partial autocorrelation function (PACF). The LSTM uses linear as the activation function, the mean square error (MSE) as the loss function and the Adam optimizer to construct the model, while the Prophet model is built on the Python platform. The root mean squared error (RMSE), mean absolute error (MAE) and Mean Absolute Percentage Error (MAPE) are used to measure the predictive performance of the model.

**Findings:**

In this research, the LSTM model had the highest prediction accuracy, followed by the Prophet model, and the SARIMA model had the lowest prediction accuracy. The trend in medical expenditure of RTIs inpatients overlapped highly with the number of RTIs inpatients.

**Conclusion:**

By adjusting the activation function and optimizer, the LSTM predicts the number of RTIs inpatients more accurately and robustly than other models. Compared with other models, LSTM models still show excellent prediction performance in the face of data with seasonal and drastic changes. The LSTM can provide a better basis for planning and management in healthcare administration.

**Implication:**

The results of this research show that it is feasible to accurately forecast the demand for healthcare resources with seasonal distribution using a suitable forecasting model. The prediction of specific medical service volumes will be an important basis for medical management to allocate medical and health resources.

## Introduction

### Background

Deaths and injuries from road traffic accidents (RTAs) are a serious global public health problem. According to the World Health Organization, more than 1.35 million people died from road traffic injuries (RTIs) worldwide in 2018. Notably, RTAs fatalities in developing countries are more than three times higher than in developed countries (1). Reducing RTAs and fatalities in developing countries has become a major common concern in the field of road traffic safety and public health worldwide (2).

As the world's most populous developing country, China has experienced a rapid expansion of its road network and the number of private cars in the last decade (2). At the same time, the incidence of RTAs is increasing yearly (3, 4). In 2003, the National Road Traffic Safety Law was introduced and implemented by the Chinese government to effectively manage transport and kerb the rise in RTAs (5). The enactment of this law has improved the traffic situation in China to a large extent, but a large number of RTIs still occur each year. According to data released by the National Bureau of Statistics, there were 247,646 RTAs nationwide in 2019, with 62,763 fatalities and 256,101 injuries, resulting in direct property damage of RMB 134 million (6).

### Motivation and objectives of the research

It is clear that China is facing a huge challenge posed by the RTAs. This challenge comes from two main sources. Firstly, there is the challenge of transport security, where the large number of RTAs is seriously undermining China's transport efficiency (7, 8). On the other hand, the drain on healthcare funding from RTIs is testing the ability of China's healthcare administration to allocate healthcare resources (9). Improving the statistical accuracy and predictive accuracy of the number of RTIs is an important task in addressing these issues (10). Therefore, policymakers urgently need a reliable predicting methodology that provides decision makers with early estimates of future RTIs and resulting healthcare expenditure based on historical time series data so that they can assess the potential risks (11).

Road safety policies and interventions should be based on an accurate assessment of the RTIs burden and projections of future trends, which are often influenced by the quality of the data, the correct estimation of parameters and the correct modeling approach (12). To this end, we propose using comparative research to develop an optimal prediction model for the number of RTIs in Northeast China to provide a basis for the allocation of health care resources by the health care sector.

### Research methodology

In previous studies, various conventional methods have been applied to estimate and predict RTAs-induced mortality in China. Researchers have often used the seasonal autoregressive integrated moving average (SARIMA) to predict the time series of RTIs mortality in China (13–16). The advantage of this model is that it is simple to model and requires few data (16). It is therefore widely used in the predicting of various time series. Other researchers have used linear models, gray models, and other methods to predict deaths due to RTIs (4, 14). The results obtained from this type of research are not very accurate and can reflect limited information. In recent years, with the widespread use of machine learning techniques, more researchers have used machine learning models to research the prediction of road traffic injuries in China (17). The use of models such as extreme gradient boosting (XGBoost) (18), Elman recurrent neural network (ERNN) (12, 19), and long and short-term memory networks (LSTM) (16, 20–23) have all led to significant improvements in RTIs prediction. The introduction of machine learning techniques provides more options for predictive research in RTIs. The relatively complex modeling approach of machine learning models, however, makes it necessary to model different problems individually. Facebook Prophet (Prophet) (24, 25) is a model that has performed very brightly in many time series prediction studies in recent years and has achieved good results in the field of disease prediction. There is no research related to the application of this model to the prediction of RTIs.

### Novelty of research

The limitations of each of these studies make them of limited value as a reference for healthcare administration. Firstly, previous studies have focused on the number of deaths and mortality rates. Healthcare administration has focused more on the number of RTIs inpatients and the cost of care. Secondly, the scope of previous studies was usually defined as the whole of China, whereas the specific trends and conditions of RTIs in different regions of China vary greatly and have little practical application to different regions. This research is more informative to policy makers from a health care resource perspective than traditional studies that focus only on deaths in RTIs.

In addition, there is a lack of studies related to regions in China that are relatively economically backwards and severely aging, especially in Northeast China. In order to fill this gap in these regions, this research takes the number of RTIs inpatients in Jilin province in Northeast China as the research object and uses three time series models, SARIMA, LSTM, and Prophet, for comparative analysis to obtain the model with the most accurate prediction effect of RTIs inpatients in Jilin province. Jilin province is the most representative region in Northeast China, and using this region as the research sample is well-representative. It provides a more reliable theoretical basis for later research.

### Thesis structure

In this research, the data were first divided into training and test sets. SARIMA, Prophet, and LSTM prediction models were built using the training set data and the prediction results were obtained separately. This research compares the prediction results of the three prediction models by comparing the difference between the prediction results of the three prediction models and the test set data, and discusses the practical usefulness of the prediction models in actual healthcare resource allocation.

## Methods

### RTIs inpatients data collection

The data used in this research were obtained from inpatient data of general hospitals in Jilin Province aggregated by the Jilin Provincial Health and Wellness Commission. The data include the time of admission, the reason for admission and the healthcare expenditure of the inpatients. Patients were selected for the research by screening those whose reason for admission to the hospital was a RTAs. Data was obtained on the number of patients admitted to the hospital as a result of RTAs between 2015 and 2020 and their spending during their treatment. Data on a total of 24,885 patients admitted to hospital for RTIs were included in this research (Supplementary Material).

**Figure 1:**
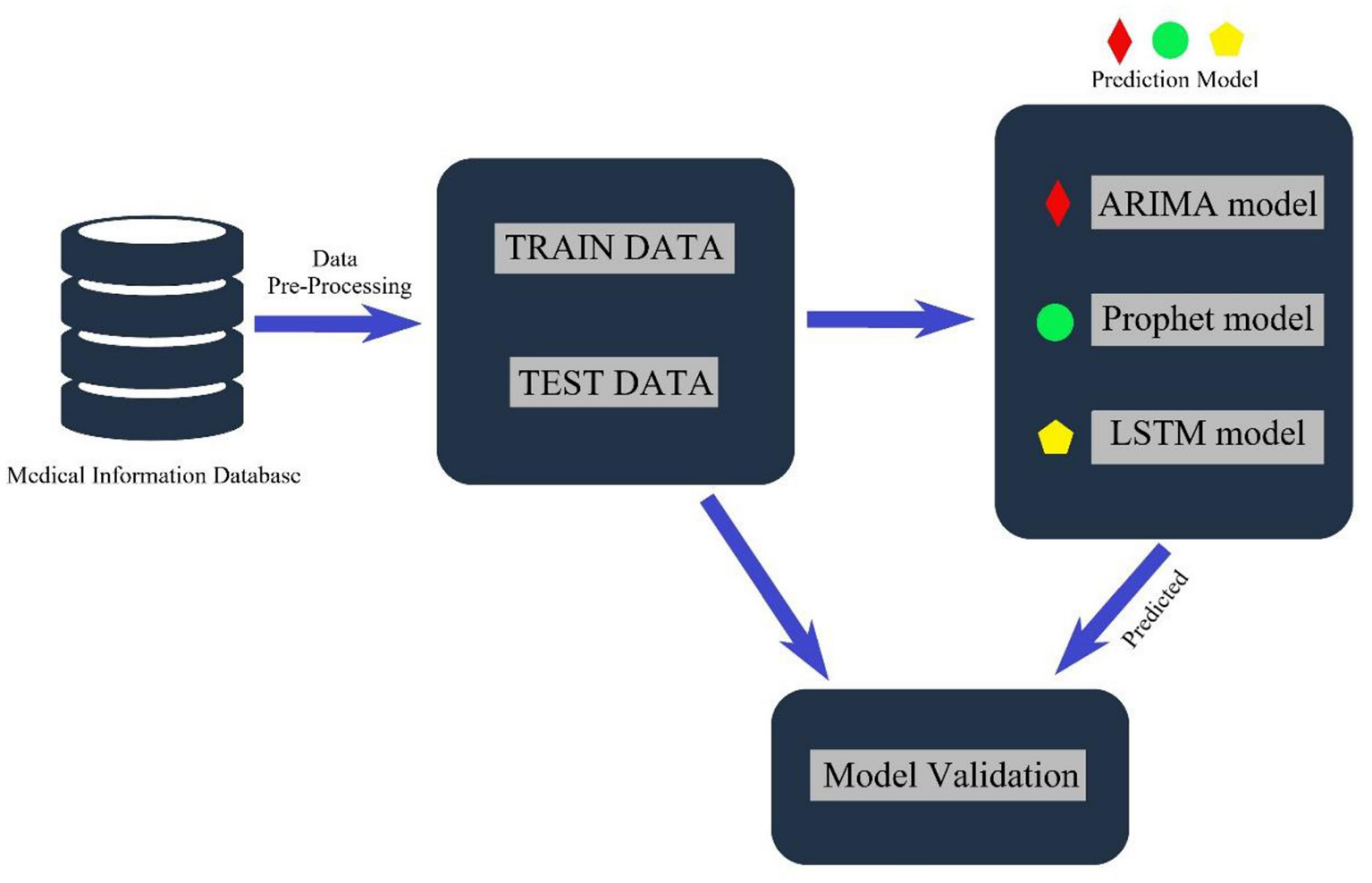
Architecture of the RTIs predictive model.

This research uses three leading models—seasonal autoregressive integrated moving average (SARIMA), Long Short-Term Memory (LSTM) and Facebook Prophet (Prophet) models—to analyze the number of RTIs inpatients. Data from 2015 to 2019 were used as the training set to train the model, and data from 2020 were used as the test set to test the model's predictive effectiveness. Figure 1 shows the typical architecture of the proposed model to predict the future count of RTIs inpatients.

### SARIMA model

ARIMA model is an Autoregressive (AR) model, moving average (MA) model and ARMA model are commonly used models in the processing of time series, and these three models are suitable for the analysis of stationary time series, but it is difficult to analyze the time series studied when there is an upwards or downwards trend. The ARIMA model, also known as Box-Jenkins model (26), is an extension of AR, MA, and ARMA models. The formula of the ARIMA model is as follows:


$$\{\begin{array}{ll} & \phi \left(B\right)\nabla^{d}x_{d}=\theta \left(B\right)\varepsilon_{t}\\ & E\left(\varepsilon_{t}\right)=0,\mathrm{Var}\left(\varepsilon_{t}\right)=\sigma_{t}^{2},E\left(\varepsilon_{t}\varepsilon_{S}\;\right)=0,s\neq t\\ & Ex_{s}\varepsilon_{t},\forall s<1 \end{array}$$

B denotes the backwards operator, and ε_*t*_ denotes the error term at time t. The model contains three parameters namely p, d, and q, p means autoregressive, d means degree of non-seasonal difference and q means order of moving average. When time series show no strong seasonal trend, the ARIMA model can predict accurately, but when time series have strong seasonal effect, the seasonal ARIMA model (SARIMA) is required (16). The formula of the SARIMA model is as follows:


$$\nabla^{d}\nabla_{S}^{D}x_{t}=\frac{\theta (B)\phi_{s}(B)}{\phi (B)\phi_{s}(B)}\varepsilon_{t}$$

Where, *x*_*t*_ denote the time series, ∇ denote the difference operation, B denote the backwards shift operator, s is the period length, and ε_*t*_ is the white noise sequence. The three parameters p, d, and q have the same meaning as the parameters in the ARIMA model, parameters P, D, Q, and s represent seasonal autoregressive, seasonal degree of difference, seasonal order of moving average and seasonal period length, respectively. We first observe the stationarity and seasonal periodicity of the time series, and then eliminate and stabilize the seasonal period of the time series by differential processing. After that, autocorrelation function (ACF) and partial autocorrelation function (PACF) pictures of the difference sequence are drawn to determine the parameters p and q of the model. We chose the model with the smallest Akaike Information Criterion (AIC) as the final model, then evaluated the fitting effect of the model by detecting the white noise of the residues.

### Facebook Prophet model

The Prophet model is an additive model for time series predicting that was open sourced by Facebook Inc. in 2017 (27). According to Google's official presentation, it works best with time series that have strong seasonal effects and several seasons of historical data (24). Prophet is robust to missing data and shifts in the trend and typically handles outliers well (28). The model quickly became a hot time series model upon its release. The model splits the time series into three main components: the seasonal term *S*_t_, the trend term *T*_t_ and the residual term *R*_t_:


$$y_{t}\;=\;S_{t}\;+\;T_{t}\;+\;R_{t}$$

Additionally, the Prophet model incorporates the effect of holiday *h*(t) to meet the needs of the actual scenario:


$$y_{t}=g\left(t\right)+s\left(t\right)+h\left(t\right)+\varepsilon_{t}$$

The model is robust to missing data and outliers and fits a wide range of data relatively well, making it a popular time series predicting model among data analysts.

### LSTM model

**Figure 2:**
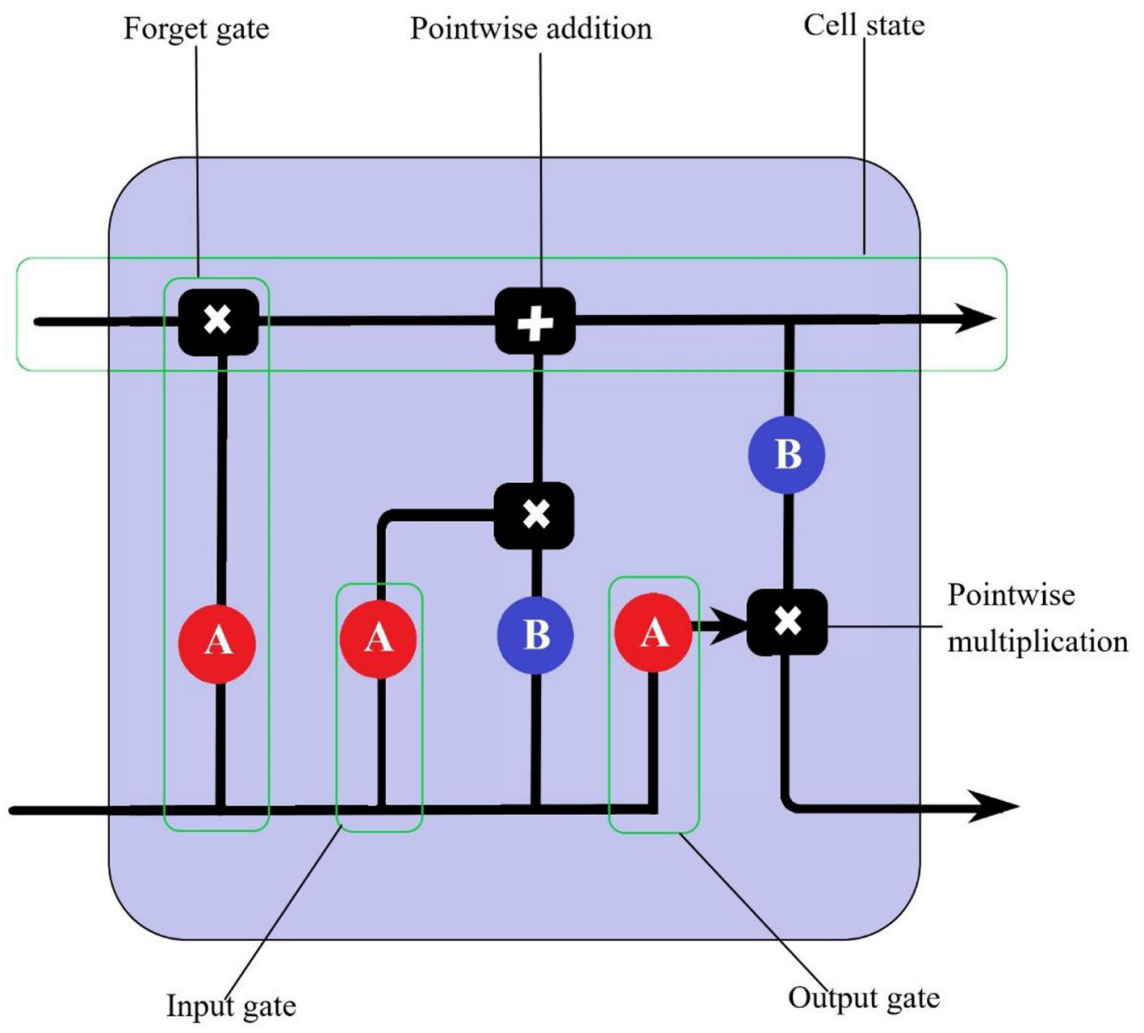
The LSTM cell consists of an input gate, an output gate and an oblivion gate. A and B are activation functions.

The LSTM model is a neural network model based on an improved RNN neural network. RNN are widely used for time series prediction, but they are hardly competent for long-term data-dependent problems (22). Hochreiter and Schmidhuber proposed the LSTM model in 1997 to improve the RNN model with memory units to overcome its limitations in long-term data dependence (29). The memory unit is self-linking, stores the network time state and is controlled by three gates: input gate, output gate and forget gate. Input gates and output gates work to control the flow of inputs and outputs from the memory unit to the rest of the network. In addition, forgetting gates are added to the memory unit, which passes output information with high weights from the previous neurone to the next neurone. LSTM neurons have memories within their pipeline that can store previous information, update the information, and pass it to the next layer or cell without losing information (Figure 2).

1. *Forgetting gate*: The function of the forgetting gate is to determine the information that needs to be retained or discarded in the middle and previous layers. The forgetting gate function can be expressed as follows:
$$\begin{array}{l} f_{t}=\sigma \left(W_{f},\cdot \;,\left[h_{t-1},,,x_{t}\right],+,b_{f}\right) \end{array}$$
2. *Input gate*: The input gate is followed by the forgetting gate, which updates the data and collates it into the storage unit by means of an activation function. The specific formula is as follows:
$$\begin{array}{l} i_{t}=\sigma \left(W_{i},\cdot \;,\left[h_{t-1},,,x_{t}\right],+,b_{t}\right) \end{array}$$
3. *Output gate*: The output gate determines the output of the model with the weight of the control state *C*_*t*_ to the current LSTM implicit layer. The initial output is obtained by the activation function and then the output values are normalized by the tanh function. The expression is as follows:
$$\begin{array}{l} o_{t}=\sigma \left(W_{o},\cdot \;,\left[h_{t-1},,,x_{t}\right],+,b_{o}\right). \end{array}$$
$$\begin{array}{l} h_{t}=o_{t}\cdot \tanh\left(C_{t}\right). \end{array}$$
4. *Memory Cell*: The memory cell uses the candidate values generated by the activation function and then updates the memory state in combination with the input information from the input gate and the current state information. The calculation formula is as follows:

$$\tilde{C_{t}}=\tanh\left(W_{c},\cdot ,\left[h_{t-1},,,x_{t}\right],+,b_{C}\right).$$

In the above formulas, σ presents activation function. W_f_, W_i_, W_c_, W_o_ denote the weight values of the forgetting gate, the input gate output gate and the memory unit. b_f_, b_i_, b_c_, b_o_ denote the deviation of each component. They are all generated by random initialization function.

A loss function is a way for a computer machine to learn the difference between the predicted and true values of a model. As machine learning models are prone to over-fitting during training, and over-fitting of the model to the input set data leads to a reduction in the generalization ability of the model. Looking at the input set and output set loss functions is the main means of determining whether a model is over-fitted (30). The input set loss function is generally considered to be larger than the output set loss function. In this research, Mean Squared Error (MSE) is used as the loss function (31) with the following equation.

$$MSE=\frac{\sum_{i=1}^{n}{\left(X_{i}-\hat{X_{i}}\right)}^{2}}{n}$$

*X*_*i*_ is the actual value, ${\hat{X}}_{i}$ is the fitting values or predicted value, i = 1…n and n is the number of observation.

### Model evaluation

Two indexes measure the prediction performance of the models: Root Mean Squared Error (RMSE), Mean Absolute Percentage Error (MAPE) and Mean Absolute Error (MAE). RMSE tends to be dominated by larger values, the MAE and MAPE give a good indication of the error between the predicted and true values (32–34).


$$RMSE=\sqrt{\frac{\sum_{i=1}^{n}{\left(X_{i}-{\hat{X}}_{i}\right)}^{2}}{n}}$$

$$MAE=\frac{\sum_{i=1}^{n}\left|X_{i}-{\hat{X}}_{i}\right|}{n}$$

$$MAPE=\frac{\sum_{i=1}^{n}\frac{\left|X_{i}-{\hat{X}}_{i}\right|}{X_{i}}\times 100}{n}$$

*X*_*i*_ is the actual value, ${\hat{X}}_{i}$ is the predicted value, i = 1…n and n is the number of observation.

### Data and analysis

Data from January 2015 to December 2019 were used as the training set for the construction of the SARIMA model and the Prophet model. Data from January 2015 to December 2018 were used as the input set January to December 2019 as the output and to train the LSTM model. MSE was used to define the loss function. The loss function of the model in the input set output set is plotted to determine if the model is over-fitted. Finally, RTIs predictions for 2020 were made using the three completed training models and compared with the true value of the number of RTIs inpatients in 2020. The accuracy of the model predictions was judged by comparing the RMSE and MAE of the three models.

Excel 2016 was used to build the monthly database of RTIs inpatients in Jilin Province, and Python 3.8.8 software was used to build the SARIMA model, LSTM model and Prophet model.

## Results

### Statistical results

**Figure 3:**
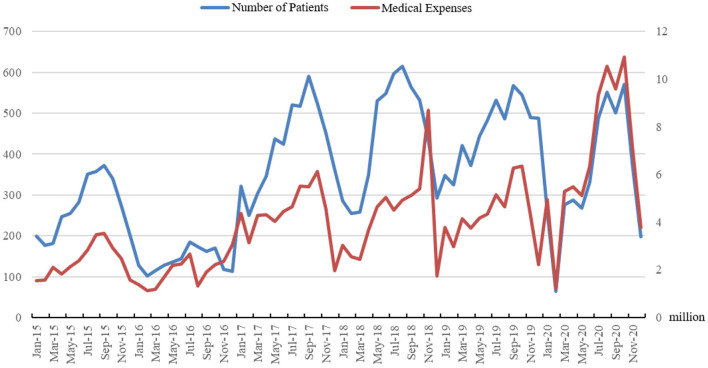
Number and healthcare expenditure of RTIs inpatients for the period 2015–2020.

As shown in Figure 3, the number of RTIs inpatients is represented by the blue line, and the red line represents the total monthly healthcare expenditure caused by RTIs inpatients. It is clear from Figure 3 that the trend in total healthcare expenditure is highly consistent with the number of inpatients. The statistics show a gradual decrease in average healthcare expenditure for RTIs inpatients between 2016 and 2019, but an increase in healthcare expenditure in 2020 due to COVID-19. The statistics show that the average healthcare expenditure of RTIs inpatients saw a large increase between 2015 and 2016. The average healthcare expenditure for RTIs inpatients gradually decreases between 2016 and 2019, before once again showing a significant rise in 2020. The medical expenditure of patients hospitalized with RTIs is reported in Table 1. The medical expenditures of patients hospitalized with RTIs vary considerably due to the degree of injury. In contrast, the mean and median medical expenditure for inpatients with RTIs did not vary significantly each year.

**Table 1:** The healthcare expenditure arising from RTIs in Jilin Province from 2015 to 2020.

	**2015**	**2016**	**2017**	**2018**	**2019**	**2020**
Maximum	935,866.8	847,102.3	868,335.4	916,696.8	566,297.9	912,172
Minimum	33.27	50.5	49.34	19	7.5	6
Mean	29,908.97	50,232.74	42,422.14	36,734.02	34,335.39	43,454.7
Q1	4,858.538	7,075.955	6,847.453	5,856.933	5,978.21	7,955.33
Median	13,152	27,712.31	22,004.19	18,998.58	18,013.31	19,147.01
Q3	39,305.65	64,587.82	52,659.97	40,473.39	49,811.57	57,307.12


### SARIMA model

A dataset of RTIs inpatients in Jilin Province in Python, using 2015 to 2019 data as the training set and 2020 data as the test set, the SARIMA model was tested for predictive effectiveness.

**Figure 4:**
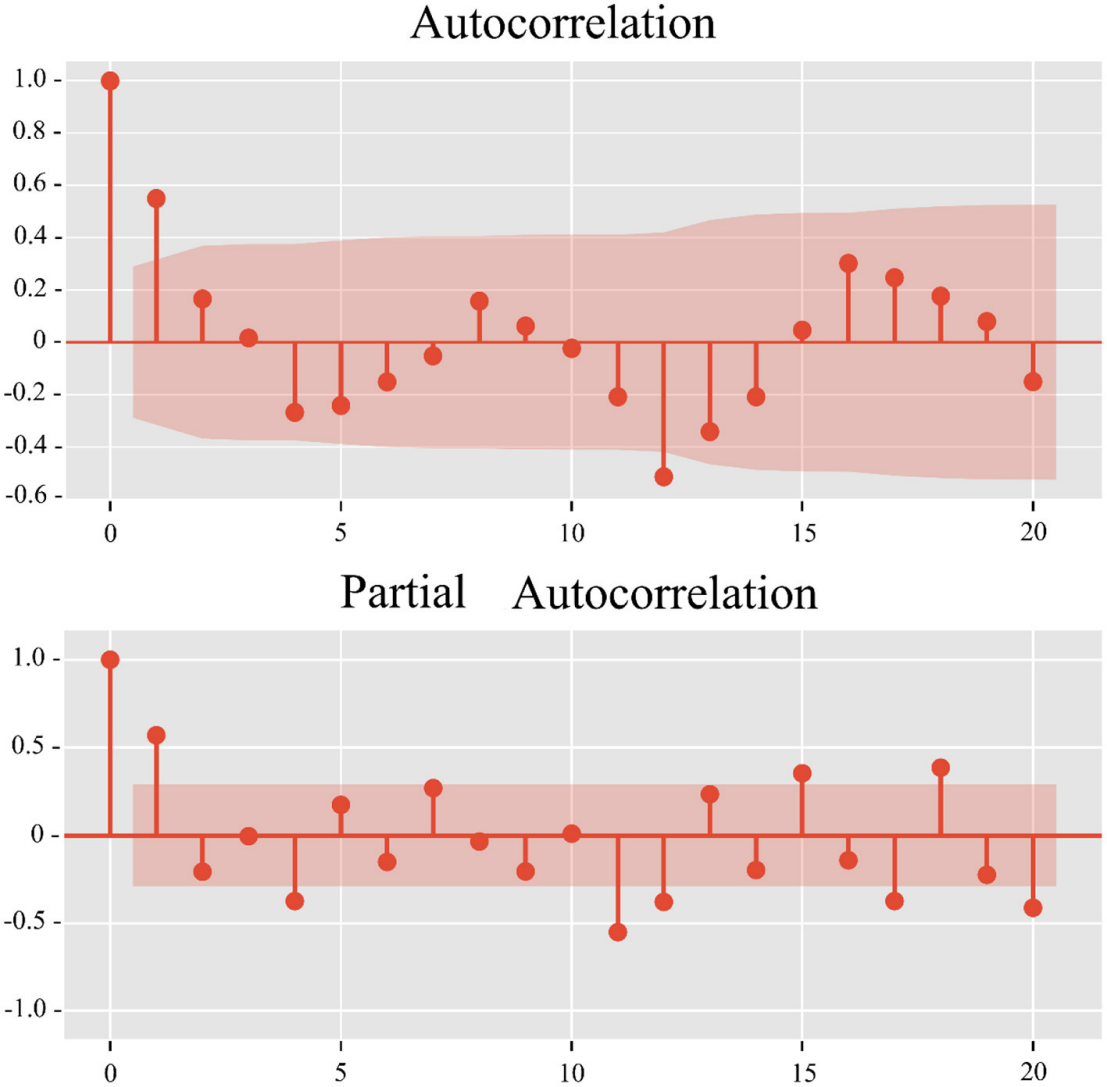
ACF and PACF images of the SARIMA (1,1,0), (2,1,3)_12_ model.

The Dickey-Fuller test was used to demonstrate that the data were non-stationary (*p* = 0.136). After first-order differencing and seasonal differencing, the SARIMA model determines the three parameters of p, d, and q from ACF and PACF images (Figure 4). The parameters of the final model are then determined by means of a minimum AIC (AIC = 101.79). The final model for the final number of road accident admissions was determined to be SARIMA (1,1,0), (2,1,3)_12_.

**Figure 5:**
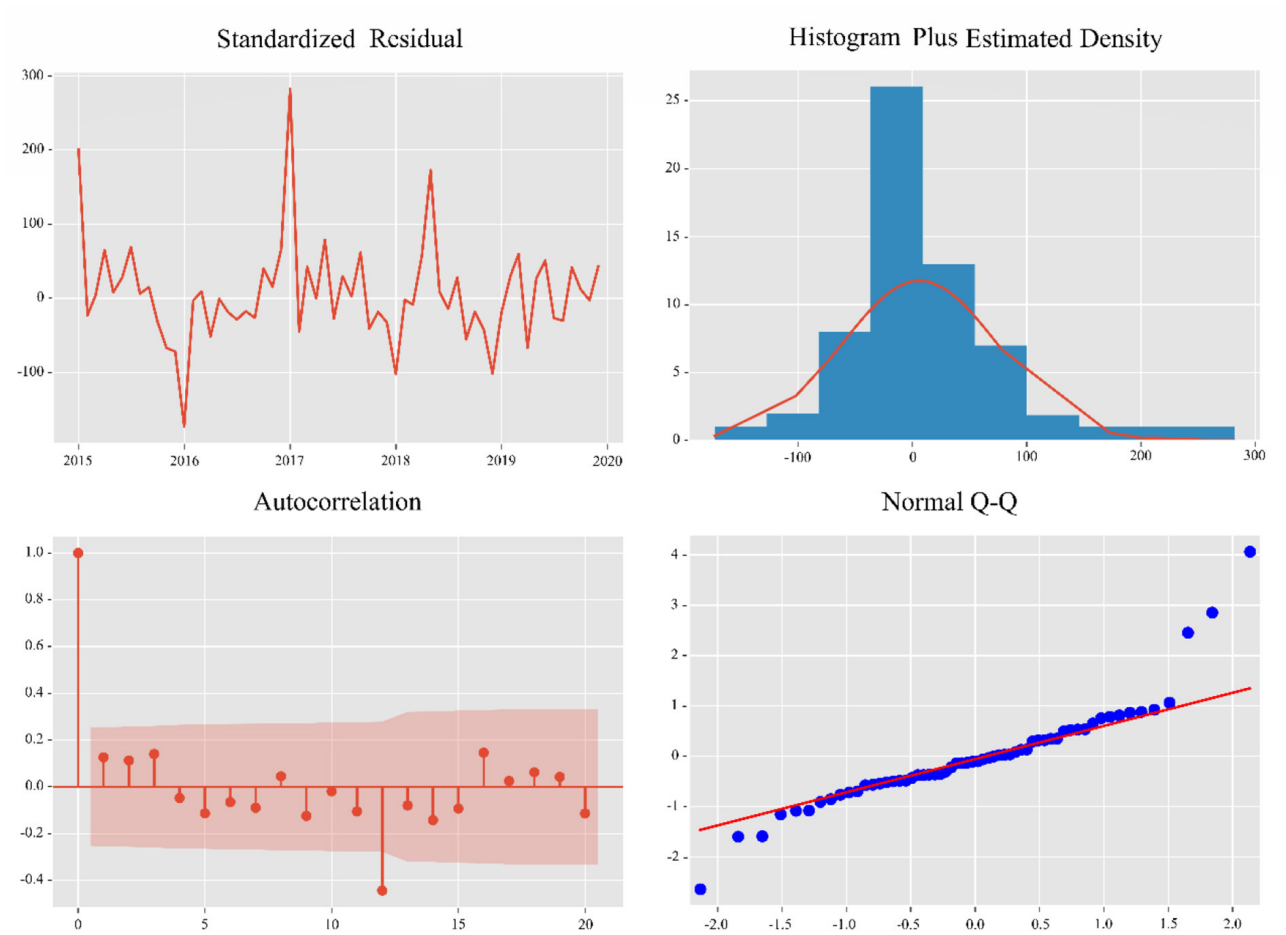
Residual analysis of the SARIMA (1,1,0),(2,1,3)_12_ model.

The Ljung-Box test was used to test whether the residuals of the model conformed to a normal distribution, with a *p*-value of 0.32. Therefore, the original hypothesis cannot be rejected (H0: residuals are normally distributed). The residuals were analyzed by plotting ACF plots of the residuals, Q-Q plots of the residuals and histograms of the residuals (Figure 5). The residuals of the model SARIMA (1,1,0), (2,1,3)_12_ models are normally distributed, which indicates that all the information in the data is extracted by the SARIMA model.

### Prophet model

This research uses the training data to build the Prophet model in Python 3.7. We implemented the trend model with a saturating growth, and the carrying capacity of the logistic growth model was set as 8.5. The change-points were automatically selected and the number of change-points was set as 25. We set the interval width as 0.8. The parameters of the Prophet model are shown in Table 2.

**Table 2:** Prophet and LSTM parameters and their values.

**Method**	**Parameters**	**Values**
LSTM	Layers	3
	No. of neurons	{16,32,64}
	Learning rate	0.01
	Dropout	0.3
	Optimizer	Adam
	Batch size	3
	Maximum Epochs	1,000
	Activation Function	Linear
Prophet	Growth	Logistic
	Changepoint Range	0.8
	Holidays	CN
	Changepoint Prior Scale	0.05

### LSTM model

The LSTM neural network was modeled using Python 3.7. To improve the training efficiency of the model, we first normalized the data before feeding it into the LSTM model. The main parameters in the LSTM model are the activation function, dropout, batch size, epoch, neurones in the hidden layer and the optimizer. The maximum number of iterations of the model is 1,000, and the model stops training when the loss function is <0.075. The parameters of the LSTM model are shown in Table 2.

**Figure 6:**
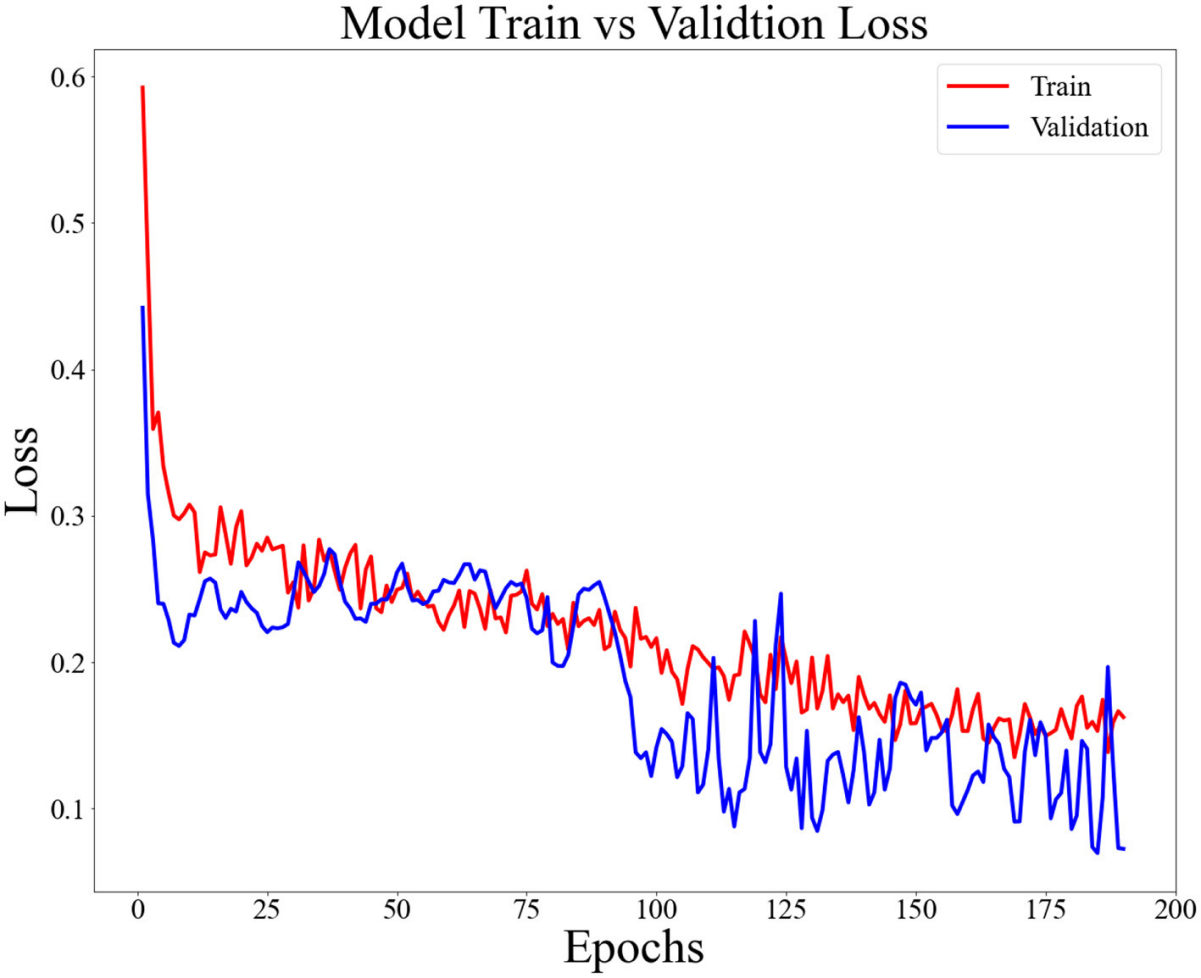
Loss function for LSTM models.

Figure 6 shows the performance of the loss functions for the training and test sets, with the red line being the training set loss function and the blue line being the test set loss function. The loss values of the training and test sets decreased at the same time. In most cases, the loss value of the training set was smaller than that of the training set. The results show that the LSTM model is well-trained and does not show any over-fitting.

### Comparison of models

**Figure 7:**
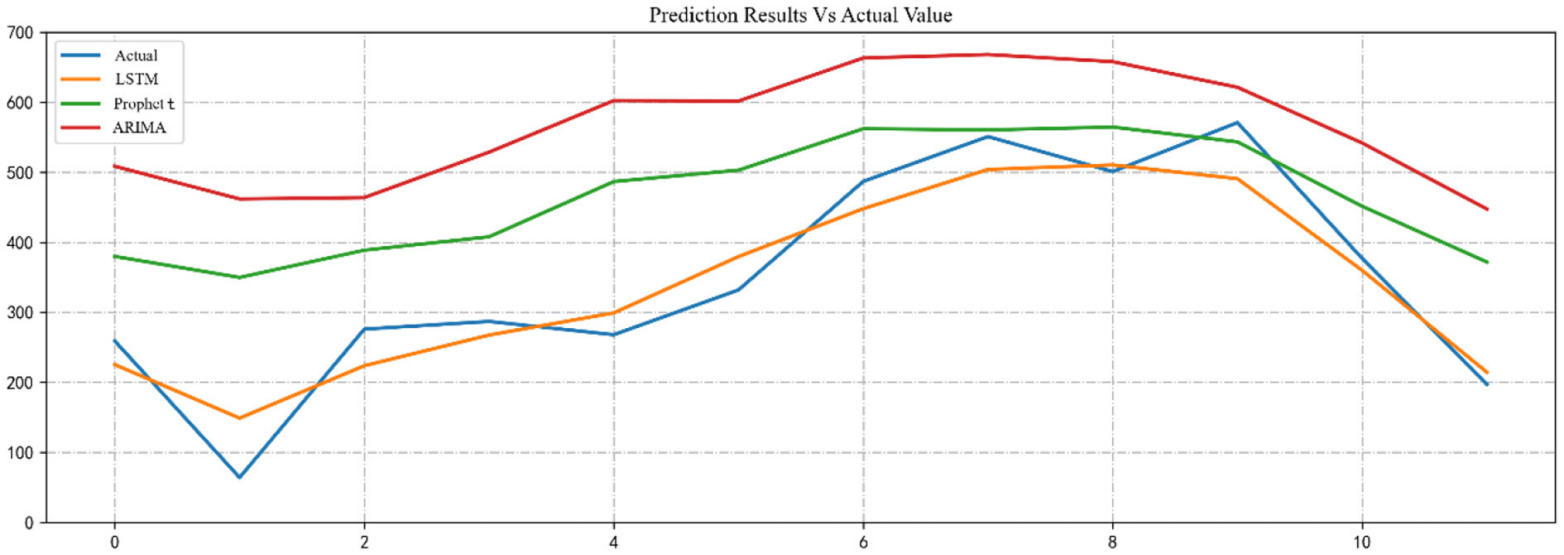
Visualizations of predicted and actual values for SARIMA, Prophet, and LSTM models.

We used the trained SARIMA, Prophet, and LSTM models to predict the number of RTIs inpatients in 2020 and compared them to the test dataset. The predictive performance of the model was evaluated by calculating the RMSE, MAE and MAPE between the three predicted and actual values. Table 3 reports the evaluation results for the SARIMA, Prophet, and LSTM models. Table 4 shows the actual values vs. the predicted values from the three models. Figure 7 visualizes the predicted and actual values of the SARIMA, Prophet, and LSTM models.

**Table 3:** LSTM, Prophet, and SARIMA prediction effect evaluation parameters.

	**LSTM**	**Prophet**	**SARIMA**
			**(1,1,0), (2,1,3)**_12_
RMSE	46.12	143.58	208.95
MAE	39.93	121.2717	191.48
MAPE	20.26%	71.04%	92.82%

**Table 4:** Actual values vs. predicted values from the three models.

**Mouth**	**Actual**	**LSTM**	**Prophet**	**SARIMA**
January	259	225.29	379.84	408.59
February	64	148.88	349.81	361.90
March	276	223.70	388.75	363.91
April	287	267.50	407.86	528.79
May	268	299.20	486.73	602.42
June	332	379.61	503.07	601.83
July	487	448.03	562.20	663.29
August	551	504.08	560.51	668.26
September	501	510.49	564.63	658.03
October	571	490.83	543.43	621.43
November	377	359.91	451.45	541.87
December	197	214.41	371.83	447.52

From these results, it can be seen that the LSTM model performs the best, the Prophet model the second best and the SARIMA model the worst.

## Discussion

Monthly trends in the number of RTIs inpatients in Jilin Province from 2015 to 2020 show a clear seasonal pattern. The number of RTIs reported from other regions in China also showed a clear seasonal pattern, but the high incidence season was different (35–38). The reasons for this difference may be the result of a combination of factors such as the length of daylight, alcohol consumption, recreational driving, and possible inclement weather (39).

Statistical analysis of health expenditure data shows that the average expenditure of RTIs inpatients changed significantly between 2015 and 2016, with the cost of treatment decreasing each year for the next 3 years. The main reason for this change is the abolition of the drug mark-up reform system that came into effect in 2016 (40, 41). The results from 2016 show that this system has succeeded in curbing the rapid increase in treatment costs. The increase in costs in 2020 is mainly due to the overall increase in healthcare expenditure as a result of the COVID-19 epidemic (42). In addition, the trend in total healthcare expenditure is highly consistent with the trend in the number of RTIs inpatients. This suggests that accurate predicting of RTIs inpatient can be a useful tool for healthcare resource planning. At the same time, the prediction of the number of patients is more in line with the needs of DRG reform in China.

This research observed a very significant decrease in the number of RTIs inpatients in Jilin Province in the first 3 months of 2020 compared to previous years. This is due to the severe travel control measures implemented in Jilin Province during this period. In the second half of 2020, as travel controls were lifted, the number of RTIs admissions gradually returned to the average for the same period in previous years. This phenomenon has been replicated in other parts of China, and management policies can have a very significant impact on RTIs incidence (43–45). The government can achieve the goal of reducing the number of RTIs occurrences by imposing reasonable regulatory measures (46, 47).

### Theoretical of predictive model

In principle, the SARIMA model has shown its effectiveness and advantages in capturing linear trends in seasonal series compared to auto-regressive integrated moving average and exponential smoothing models, and can be easily developed by many data analysis software. SARIMA is one of the most effective linear models for forecasting seasonal time series (13). However, the drawbacks of SARIMA models are also apparent. When generating a smooth time series, it is usually necessary to pre-process a large amount of longitudinal data and use appropriate transformation techniques, such as differencing and transformation, to stabilize the variables before modeling (48). Although the SARIMA model can capture linear trends in seasonal time series, it may not accurately predict RTIs because of the non-linearity of the data and the various influences associated with traffic fatalities. In contrast, the LSTM model is one of the RNN models that can approximate the ideal accuracy for complex non-linear functions of real-world data.

In this research, the advantage of the LSTM in the comparison of the three models is very clear, and the reason for this phenomenon may have a lot to do with the characteristics of the data (49–51). In previous studies using additive models such as SARIMA models and Prophet, one is often dealing with more stable data. SARIMA and Prophet models have a strong advantage in dealing with time series data with significant seasonality. However, some researchers have pointed out that these models are less resistant to disturbances (16, 52), and in this research, there was a very large drop in the data for the first 3 months of 2020, which led to a relatively large deviation of these two models. LSTM models can be adapted to more application scenarios by calling different activation functions, which also makes them more resistant to disturbances than traditional additive models, and when external factors (16). This also makes the LSTM model more resistant to disturbances than the traditional additive model, so that when external factors change significantly, the LSTM model can still make reliable predicts (21). In this research, the difference between the prediction accuracy of the LSTM model and the traditional model becomes more significant due to the large change in the number of RTIs inpatients in 2020. It is worth mentioning that the predicted values given by the three models for the second half of 2020 are closer to the true values. This provides some evidence that the SARIMA and Prophet models are able to obtain satisfactory prediction results in a stable seasonal time series.

### Advantages and limitations

Admittedly, there are some limitations to this research. Firstly, the data for this research was sourced from the healthcare finance system of the Jilin Provincial Health and Wellness Commission. The data from this system is derived from hospital reporting and there may be selection or omission bias, which may affect the accuracy of the predictions. Secondly, our research focuses on the north-eastern Chinese city of Jilin, and the results obtained in this research are of considerable referable value in areas with similar natural, social and environmental factors. However, the reference value is limited for regions where natural, social and environmental factors differ significantly. Finally, although the LSTM model has obvious advantages in terms of prediction accuracy, the training time and modeling complexity of this model are much greater than those of the other two models.

### Policy recommendations

Based on this, we propose the following policy recommendations: (1) Introducing effective traffic control policies or optimizing the urban traffic layout by the relevant authorities can achieve efficient traffic flow. (2) Improving the infrastructure of urban traffic and improving the possibility of roads can effectively reduce RTAs. (3) Establishing green channels can improve the speed of handling RTIs inpatients. The timely treatment of people injured in an accident not only improves the effectiveness of treatment but also reduces the corresponding healthcare expenditure. (4) Analyzing the high incidence of RTAs in cities through the use of big data tools and carrying out targeted transformation and diversion of these areas can reduce the number of RTAs. This research demonstrated that the LSTM model can accurately predict the number of RTIs inpatients in Jilin Province. This suggests that we can use this model to effectively predict the demand for services in different subgroups of the DRG in Jilin Province in the future so that during the process of DRG reform, a more scientific and effective budget allocation can be made.

## Conclusion

By adjusting the activation function and optimizer, LSTM predicts the number of inpatient RTIs more accurately and more robustly than other models. Compared with other models, LSTM models still show excellent prediction performance in the face of data with seasonal and drastic changes. Proper use of LSTM model can provide a better basis for planning and management by healthcare administrations. As China is the largest developing country in the world, the present research results are of strong value to developing countries in similar situations.

## Data availability statement

The original contributions presented in the study are included in the article/supplementary material, further inquiries can be directed to the corresponding author.

## Author contributions

TF and ZZ design research and draft article. Data collection and analysis were done by JX and MLiu. MLi and HJ revised the article and put forward suggestions. All authors commented on previous versions of the manuscript and read and approved the final manuscript.

## Conflict of interest

The authors declare that the research was conducted in the absence of any commercial or financial relationships that could be construed as a potential conflict of interest.

## Publisher's note

All claims expressed in this article are solely those of the authors and do not necessarily represent those of their affiliated organizations, or those of the publisher, the editors and the reviewers. Any product that may be evaluated in this article, or claim that may be made by its manufacturer, is not guaranteed or endorsed by the publisher.
